# Implementation of the 2015 European Society of Cardiology guidelines for the management of infective endocarditis in the Netherlands

**DOI:** 10.1007/s12471-020-01489-9

**Published:** 2020-09-09

**Authors:** A. R. Wahadat, J. W. Deckers, R. P. J. Budde, J. T. M. van der Meer, E. H. Natour, J. ten Oever, A. L. J. Kortlever-van der Spek, B. H. Stegeman, N. J. Verkaik, J. W. Roos-Hesselink, W. Tanis

**Affiliations:** 1 Department of Cardiology, Thoraxcenter, Erasmus Medical Centre, Rotterdam, The Netherlands; 2 Department of Radiology and Nuclear Medicine, Erasmus Medical Centre, Rotterdam, The Netherlands; 3 Department of Cardiology, Haga Teaching Hospital, The Hague, The Netherlands; 4 Department of Internal Medicine and Infectious Diseases, AmsterdamUMC, location AMC, Amsterdam, The Netherlands; 5 Department of Thoracic Surgery, Maastricht University Medical Centre, Maastricht, The Netherlands; 6 Department of Internal Medicine and Radboud Centre for Infectious diseases, Radboud University Medical Centre, Nijmegen, The Netherlands; 7 Knowledge Institute of the Federation of Medical Specialists, Utrecht, The Netherlands; 8 Department of Medical Microbiology and Infectious Diseases, Erasmus Medical Centre, Rotterdam, The Netherlands

**Keywords:** Infective endocarditis, Microbiological diagnosis, Disease management, Cardiac imaging, Infection, Prosthetic heart valves

## Abstract

Because the occurrence of infective endocarditis (IE) continues to be associated with high mortality, a working group was created by the Dutch Society of Cardiology to examine how the most recent European Society of Cardiology (ESC) guidelines for IE management could be implemented most effectively in the Netherlands. In order to investigate current Dutch IE practices, the working group conducted a country-wide survey. Based on the results obtained, it was concluded that most ESC recommendations could be endorsed, albeit with some adjustments. For instance, the suggested pre-operative screening and treatment of nasal carriers of *Staphylococcus aureus* as formulated in the ESC guideline was found to be dissimilar to current Dutch practice, and was therefore made less restrictive. The recently adapted ESC diagnostic criteria for IE were endorsed, while the practical employment of the relevant diagnostic techniques was simplified in an adapted flowchart. In addition, the presence of a multidisciplinary, so-called ‘endocarditis team’ in tertiary centres was proposed as a quality indicator. An adapted flowchart specifically tailored to Dutch practice for microbiological diagnostic purposes was constructed. Lastly, the working group recommended the *Stichting Werkgroep Antibioticabeleid* (SWAB; Dutch Working Party on Antibiotic Policy) guidelines for IE treatment instead of the antibiotic regimens proposed by the ESC.

## Background and introduction

One of the oldest cardiac diseases, infective endocarditis (IE), remains one of the most fatal manifestations of heart disease [1]. Despite considerable progress in diagnosis and treatment, the in-hospital mortality of IE continues to be about 20%, essentially unchanged during the past decades [2].

The importance of IE is reflected in the frequent publication of new guidelines, for instance by the European Society of Cardiology (ESC) [1]. In 2017, the Dutch Society of Cardiology created a working group—funded by the Quality Foundation of the Dutch Medical Specialists (SKMS)—to investigate whether and how the recommendations summarised in the most recent ESC guidelines on IE could be implemented most effectively in the Netherlands. To investigate current Dutch IE practices, the working group conducted a short country-wide survey. The medical topics raised in the survey are presented in Tab. 1. This report summarises the findings and recommendations of the working group.

**Table 1:** Selection of recommendations by the working group with regard to the European Society of Cardiology (*ESC*) guidelines. *SWAB* *Stichting Werkgroep Antibioticabeleid* (Dutch Working Party on Antibiotic Policy)

Topic	Recommendations in ESC guidelines	Recommendation by the working group
Antibiotic prophylaxis	Reserve antibiotic prophylaxis for high-risk individuals undergoing dental procedures	No change or comment by the working group
Prevention of infection before cardiac or vascular interventions	Screen every patient and treat *Staphylococcus aureus* carriers only pre-operatively	Pre-operative screening and/or treatment of nasal carriage of *Staphylococcus aureus *is recommended before elective surgery in order to treat carriers^a^
Microbiological diagnosis	Use the recommendation as presented in the ESC guidelines	Use flowchart as presented in Fig. 1 ^a^
Diagnostic imaging and criteria	Use diagnostic ESC criteria and the recommendation presented in the guidelines	Use diagnostic ESC criteria and the flowchart as presented in Fig. 2 ^a^
Endocarditis team	Centres without cardio-thoracic facilities must consult the regional endocarditis team in cases of (suspected) IE	No change or comment by the working group
Antimicrobial therapy	Antimicrobial therapy according to the ESC guidelines	Antimicrobial therapy according to SWAB guidelines^a^
Surgery	Indication and timing of surgery as presented in the guidelines	No change or comment for the indication of surgery Timing of surgery determined by the specialists involved^a^
Discharge	Transthoracic echo after completion of therapy Regular follow-up including blood samples Good oral health maintenance	No change or comment by the working group

^a^Different recommendation made by the working group compared with the ESC guidelines

## Prevention and prophylaxis

Although widespread antibiotic prophylaxis for IE has long been considered effective, the policy for liberal use of antibiotic prophylaxis has gradually changed to more restricted indications. Of note, the 2008 guidelines of the National Institute of Health and Clinical Excellence (NICE) from the UK recommended that antibiotic prophylaxis should be abandoned completely [3]. However, this recommendation was revised after a patient with aortic valve prosthesis died from IE after undergoing a dental procedure without—in line with the NICE guidelines—the use of prophylaxis. The NICE guidelines from 2016 recommend dentists to inform the patient about the level of risk and let him or her decide whether or not to receive antibiotic prophylaxis [4]. The strategy currently endorsed by the American College of Cardiology (ACC), American Heart Association (AHA) and ESC reserves antibiotic prophylaxis for individuals with cardiac disease at high risk of IE, e.g. for patients with a prosthetic valve, a history of IE, or with cyanotic congenital heart disease undergoing a dental procedure with a high risk of bacteraemia (usually involving perforation of the gingiva) [1, 5]. Of course, proper oral hygiene is strongly promoted universally. The working group decided to endorse the recommendations of the ESC guidelines on prophylaxis in high-risk subjects without changes or comments [1].

Nasal carriers of *Staphylococcus aureus *have more infections after cardiac surgery [6], and the pre-operative eradication of this micro-organism is thus important. To this end, two options are available. In the first, all subjects—without additional testing—are treated locally with an antibiotic ointment, usually mupirocin. Another option is to screen every patient, and to treat *S. aureus *carriers only. The ESC guidelines recommend only the latter procedure. But the merits of the two methods are of course comparable, and this is—according to the survey—reflected by the concomitant use of both approaches for decolonisation of *S. aureus* in Dutch hospitals. Therefore, the text of the recommendation in Tab. 7 of the guidelines was (slightly) adapted as follows: ‘Preoperative screening and/or treatment of nasal carriage of *Staphylococcus aureus *is recommended before elective cardiac surgery in order to treat carriers’, while the last recommendation of the same table (‘Systemic local treatment without screening of *Staphylococcus aureus*’ is not recommended) was deleted.

### Microbiological diagnosis

**Fig. 1:**
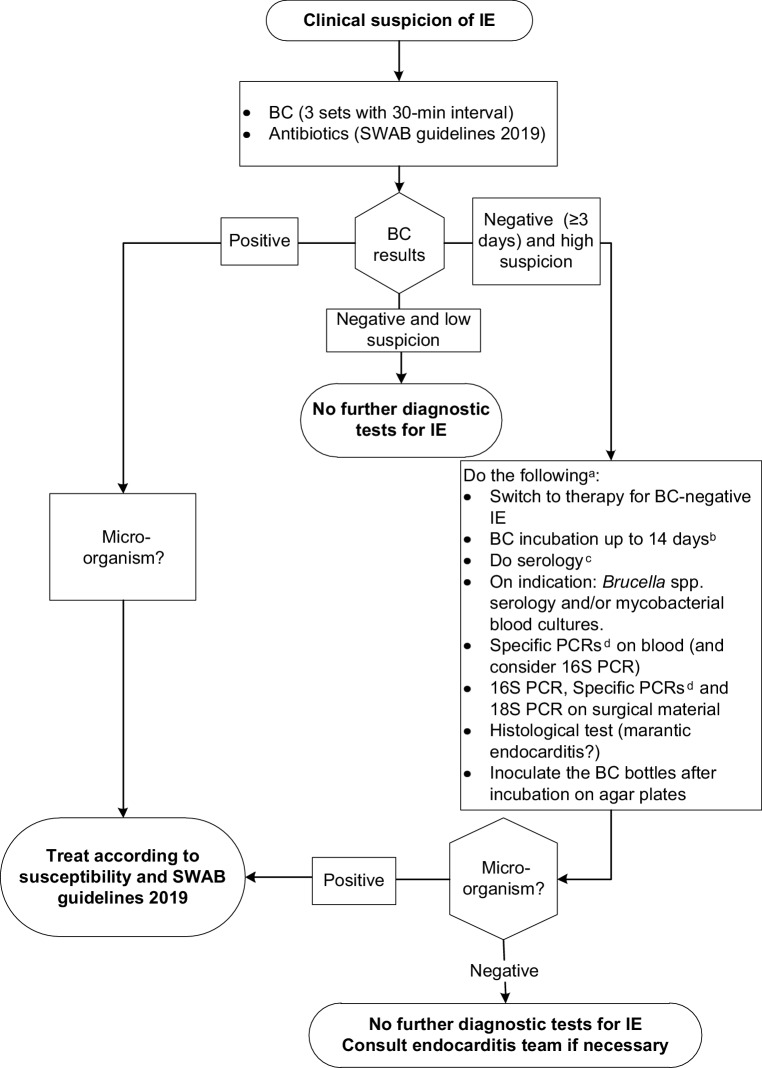
Flowchart of microbiological tests for infective endocarditis in The Netherlands. *IE* Infective endocarditis, *BC* blood cultures, *SWAB* *Stichting Werkgroep Antibiotica Beleid* (Dutch Working Party on Antibiotic Policy), *PCR* polymerase chain reaction. ^a^If the diagnostic test is not available, send the blood samples and/or blood cultures to a reference laboratory. ^b^So as not to miss *Cutibacterium acnes* and/or if blood cultures were drawn while receiving antimicrobial therapy. ^c^*Bartonella* spp. (IgM, IgG), *Coxiella burnetii* (including indirect immunofluorescent assay phase I IgG),* Legionella* spp. (IgM, IgG), *Mycoplasma *spp. (IgM, IgG). ^d^Specific PCRs: *Bartonella* spp., *Coxiella burnetii*, *Legionella* spp., *Mycoplasma* spp., *Tropheryma whipplei*

**Fig. 2:**
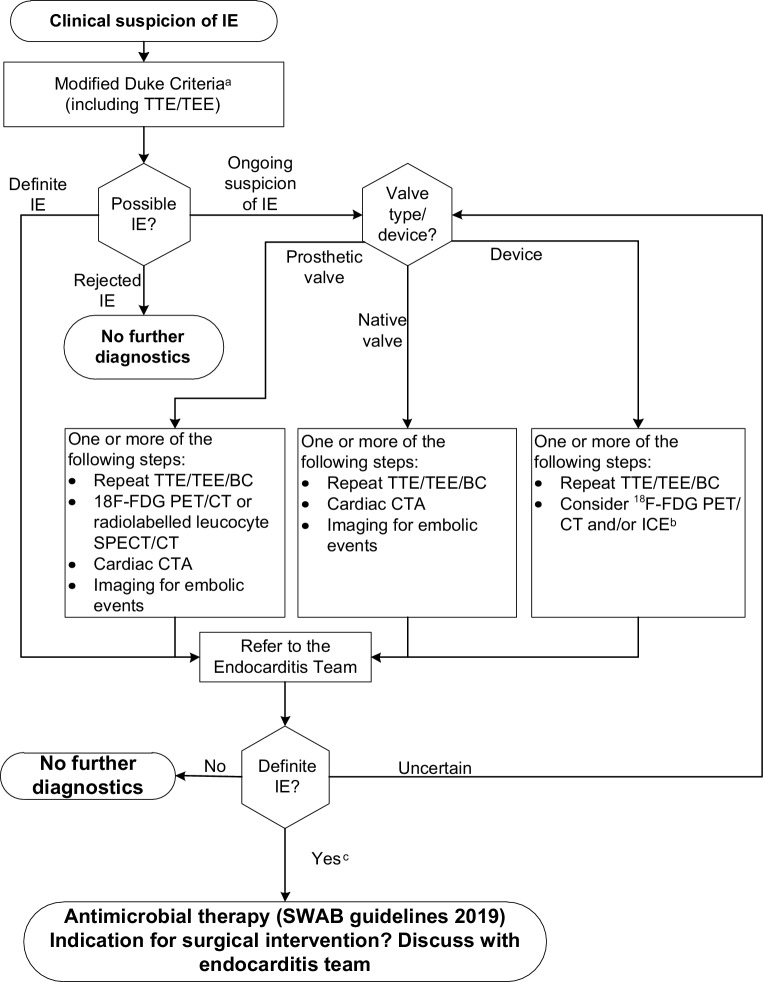
Flowchart of diagnostic imaging for infective endocarditis in the Netherlands. *IE* infective endocarditis, *TTE* transthoracic echocardiogram, *TEE* transoesophageal echocardiogram, *BC* blood cultures, ^*18*^*F‑FDG PET/CT* ^18^F‑fluorodeoxyglucose positron emission tomography computed tomography, *SPECT* single photon emission computed tomography, *CTA* computed tomography angiography, *ICE* intracardiac echocardiogram, *SWAB* *Stichting Werkgroep Antibiotica Beleid* (Dutch Working Party on Antibiotic Policy). ^a^[1, 25]. ^b^[26]. ^c^Consider referring to a tertiary referral centre when there is definite IE and one or more of the following: congenital heart disease in pregnancy, prosthetic valve endocarditis, heart failure, perivalvular extension or uncontrolled infection, embolic events or cerebrovascular accident, arrhythmia or conduction disturbances

Positive blood cultures remain the cornerstone of IE diagnosis. At least three sets with sufficient volume should be taken at 30-min intervals, and sampling preferably be obtained from a peripheral vein. When a micro-organism has been identified and appropriate antimicrobial treatment is commenced based on susceptibility results, blood cultures should be repeated every 48–72 h until blood cultures remain sterile to verify the effectiveness of the therapeutic regimen. Blood-culture-negative IE refers to IE in which no causative micro-organism can be identified using standard culture methods. In such instances, bacteria such as *Bartonella* spp. or *Coxiella burnetii*, fungi or fastidious bacteria may be in play and additional diagnostic testing may be required. Table 12 and Fig. 2 in the ESC guidelines refer to these circumstances. However, some microbiological tests included therein are not available in the Netherlands. The working group has therefore developed a flowchart adapted to Dutch practice (Fig. 1).

## Diagnostic imaging and criteria

While the modified Duke criteria, which rely heavily on positive blood cultures and findings compatible with IE at echocardiography [7], remain the mainstay for diagnosing IE, current guidelines reflect the increasing importance of more advanced imaging techniques [1]. In particular, computed tomography (CT), positron emission tomography with CT (PET-CT) and magnetic resonance imaging have emerged as valuable additional imaging techniques that provide complementary diagnostic information to echocardiography [1]. Available data—also from the Netherlands—indicate increased diagnostic accuracy when these techniques are added to the modified Duke criteria, especially in prosthetic valve endocarditis (PVE) [8–10]. The guidelines provide detailed recommendations on the use of various imaging techniques in both native valve IE and PVE, as well in the diagnosis of cardiac-device-related endocarditis [7, 8]. The working group has combined the text and figures that describe these recommendations in the ESC guidelines into a single scheme (Fig. 2).

**Fig. 3:**
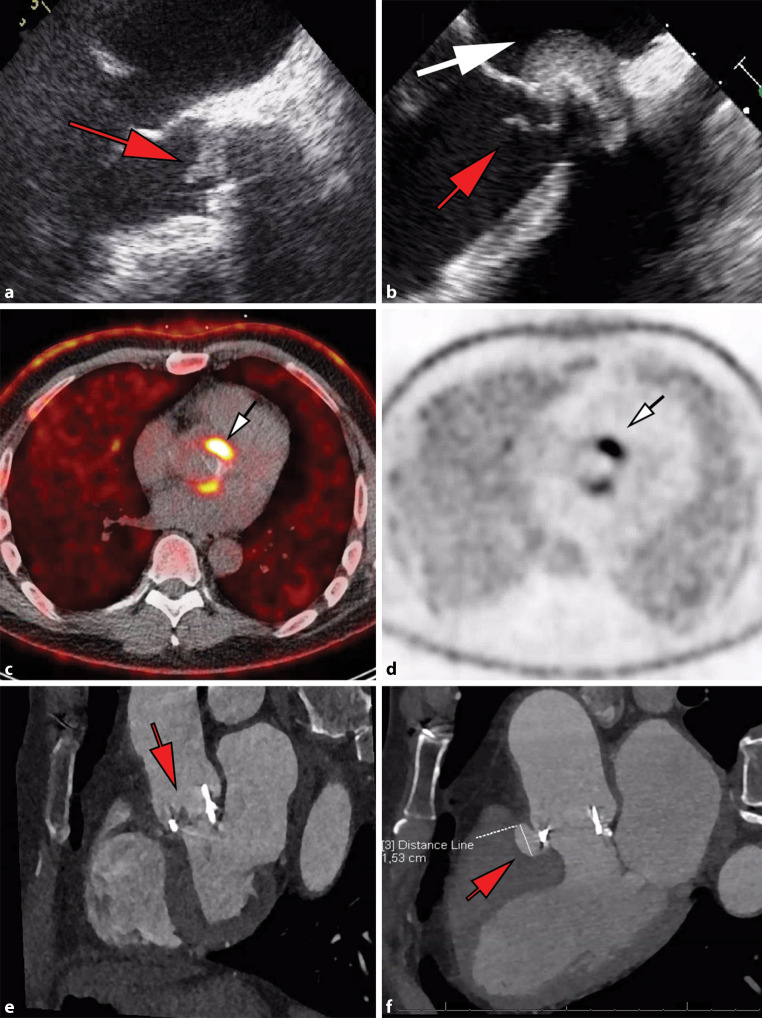
Different examples of major imaging diagnostic criteria: two cases of positive transoesophageal echocardiogram (**a**, **b**), one case of positive ^18^F‑FDG PET/CT (**c**, **d**) and one case of positive cardiac CT (**e,** **f**). **a** A case of a mechanical aortic valve with signs of vegetation (*red arrow*). The *red arrow* in **b** also indicates a vegetation on the aortic valve bioprosthesis, whereas the *white arrow* indicates a possible abscess of the aortic root. In **c** (fused PET/CT images) and in **d** (non-attenuated PET images) the *white arrows* indicate ^18^F‑FDG uptake around the aortic valve bioprosthesis as a sign of possible infection. The *red arrow* in **e** indicates a vegetation on one of the leaflets of an aortic valve bioprosthesis. Finally, the *red arrow* in **f** indicates a mycotic aneurysm alongside the aortic valve bioprosthesis

The diagnostic accuracy of the modified Duke criteria—which merge the presence of an infective syndrome and endocardial involvement, classically employing echocardiography—is only moderate, in particular in IE of a prosthetic valve [1, 11, 12]. Advanced imaging techniques—as described above—may not only be helpful in the detection of endocardial lesions when added to echocardiography, but also in establishing the presence of (clinically silent) vascular phenomena such as embolic events and infectious aneurysm [13]. Acknowledging this, the most recent ESC guidelines have added the identification of paravalvular lesions by CT and, in the setting of PVE, abnormal activity near the site of the prosthesis on ^18^F‑fluorodeoxyglucose (^18^F‑FDG) PET/CT or radiolabelled leucocyte single photon emission computed tomography (SPECT)/CT, as a ‘major’ criterion for IE. The currently applicable two major and five minor criteria for IE are described in Tab. 2. Examples of positive echocardiogram, ^18^F‑FDG PET/CT and cardiac CT are demonstrated in Fig. 3.

**Table 2:** Diagnostic criteria for infective endocarditis (*IE*) according to the 2015 European Society of Cardiology (*ESC*) guidelines for IE

*Major criteria*
1. Blood cultures positive for IE a. Typical micro-organisms consistent with IE from 2 separate blood cultures: – Viridans streptococci, *Streptococcus gallolyticus* (*Streptococcus bovis*), HACEK group^a^, *Staphylococcus aureus*; or – Community-acquired enterococci, in the absence of a primary focus; or b. Micro-organisms consistent with IE from persistently positive blood cultures: – ≥2 positive blood cultures of blood samples drawn >12 h apart; or – All of 3 or a majority of ≥4 separate cultures of blood (with first and last samples drawn ≥1 h apart); or c. *Coxiella burnetii* phase I IgG antibody titre >1:1024
2. Imaging positive for IE a. Echocardiogram positive for IE: – Vegetation – Abscess, pseudoaneurysm, intracardiac fistula – Valvular perforation or aneurysm – New partial dehiscence of prosthetic valve b. Abnormal activity around the site of prosthetic valve implantation detected by ^18^F‑FDG PET/CT (only if the prosthesis was implanted for >3 months) c. Paravalvular lesions and/or vegetation detected by cardiac CTA
*Minor criteria*
1. Predisposing heart condition or injection drug use 2. Fever defined as temperature >38 °C 3. Vascular phenomena (including those detected by imaging only) 4. Immunological phenomena (e.g. Janeway lesions, Osler’s nodes) 5. Positive blood culture but does not meet a major criterion as noted above or serological evidence of active infection with organism consistent with IE
Definite IE – Clinical criteria: 2 major or 1 major +3 minor or 5 minor criteria – Pathological criteria: microorganism cultures from the vegetation or confirmed by histological examination of vegetation/intra-cardiac abscess showing active endocarditis Possible IE – Clinical criteria: 1 major +1 minor or 3 minor criteria

Data partially derived from the 2015 ESC guidelines for IE [1]

*IE* infective endocarditis, ^*18*^*F‑FDG* ^18^F‑fluorodeoxyglucose, *PET/CT* positron emission tomography/computed tomography, *CTA* computed tomography angiography

^a^*Haemophilus, Aggregatibacter, Cardiobacterium, Eikenella, Kingella*

### The ‘endocarditis team’

An important addition in the latest ESC guidelines is the recommendation to establish a multidisciplinary ‘endocarditis team’. Such a team, comprising—at least—a cardiologist, cardio-thoracic surgeon, infectious diseases specialist, microbiologist and radiologist/nuclear medicine physician should provide the expertise needed to treat complex IE patients. The guidelines refer—among other things—to the team approach adopted in France, with standardised medical therapy and uniform recommendations for surgical interventions that were found to improve outcome relative to earlier experience [14–18]. A comparable recommendation has been made in the AHA/ACC guidelines for the management of patients with valvular disease [19]. In line with the ESC guidelines, the working group recommended that each of the current 16 Dutch tertiary referral centres with cardio-thoracic facilities create a specific regional endocarditis team. Moreover, the working group proposed to qualify the presence and composition of such a team as a quality indicator. The working group endorsed the ESC guidelines recommendation that centres without cardio-thoracic facilities must consult the regional endocarditis team in cases of (suspected) IE.

## Antimicrobial therapy

There are major differences between European countries in the use of antimicrobial therapy and consequently in the antibiotic resistance patterns of pathogens. The Netherlands has the lowest rate of antibiotic use in Europe. The result is a stable level of antimicrobial resistance, whereas most countries experience increasing levels each year. European guidelines for antimicrobial therapy therefore cannot be simply adhered to but have to be tailored to individual countries. Recommendations for antibiotic therapy in the Netherlands are provided by the *Stichting Werkgroep Antibiotica Beleid* (SWAB; Dutch Working Party on Antibiotic Policy). Importantly, SWAB recently updated their guidelines for antibiotic treatment for IE, on the basis of an in-depth comparison of the most recent ESC and the AHA IE guidelines. In cases of discordance between the recommendations in these documents, SWAB guidance is based on a formal literature review on best current Dutch practice, taking into consideration national resistance patterns and dosing habits. For all these reasons, the working group recommended the employment of the SWAB guidelines for subsequent use in the Netherlands [20].

## Main complications and their management

Heart failure resulting from valvular regurgitation or obstruction, uncontrolled infection and embolic events occurring under adequate antibiotic treatment constitute major complications of IE and may require surgical treatment [1, 21–24]. The working group endorsed the ESC indications for cardiac surgery without modifications. However, the timing of the surgical procedure was left to the discretion of the specialists involved. In accordance with the recommendations of the ESC guidelines, complex IE patients should be referred early to a regional centre with cardio-thoracic facilities. Such cases include, but are not limited to, IE patients with congenital heart disease, PVE, pregnant women, patients with heart failure, uncontrolled infection, rhythm abnormalities or stroke.

## References

[1] Habib G, Lancellotti P, Antunes MJ (2015). 2015 ESC Guidelines for the management of infective endocarditis: the Task Force for the Management of Infective Endocarditis of the European Society of Cardiology (ESC). Endorsed by: European Association for Cardio-Thoracic Surgery (EACTS), the European Association of Nuclear Medicine (EANM). Eur Heart J.

[2] Habib G, Erba PA, Iung B (2019). Clinical presentation, aetiology and outcome of infective endocarditis. Results of the ESC-EORP EURO-ENDO (European infective endocarditis) registry: a prospective cohort study. Eur Heart J.

[3] Centre for Clinical Practice at NICE (UK).. Prophylaxis against infective endocarditis: antimicrobial prophylaxis against infective endocarditis in adults and children undergoing interventional procedures. 2008. https://www.nice.org.uk/guidance/cg64, updated Mar: Infective endocarditis in adults and children undergoing interventional procedures. 2008. Accessed: 3 June 2020. 21656971

[4] Thornhill MH, Chambers JB, Dayer M, Shanson D (2016). A change in the NICE guidelines on antibiotic prophylaxis for dental procedures. Br J Gen Pract.

[5] Duval X, Hoen B (2015). Prophylaxis for infective endocarditis: let’s end the debate. Lancet.

[6] Bode LGM, Kluytmans JAJW, Wertheim HFL (2010). Preventing surgical-site infections in nasal carriers of Staphylococcus aureus. N Engl J Med.

[7] Tanis W, Teske AJ, van Herwerden LA (2015). The additional value of three-dimensional transesophageal echocardiography in complex aortic prosthetic heart valve endocarditis. Echocardiography.

[8] Wahadat AR, Tanis W, Swart LE (2019). Added value of (18)F-FDG-PET/CT and cardiac CTA in suspected transcatheter aortic valve endocarditis. J Nucl Cardiol.

[9] Mahmood M, Kendi AT, Ajmal S (2019). Meta-analysis of 18F-FDG PET/CT in the diagnosis of infective endocarditis. J Nucl Cardiol.

[10] Tanis W, Budde RPJ, van der Bilt IAC (2016). Novel imaging strategies for the detection of prosthetic heart valve obstruction and endocarditis. Neth Heart J.

[11] Habib G, Hoen B, Tornos P (2009). Guidelines on the prevention, diagnosis, and treatment of infective endocarditis (new version 2009): the Task Force on the Prevention, Diagnosis, and Treatment of Infective Endocarditis of the European Society of Cardiology (ESC). Endorsed by the European Society of Clinical Microbiology and Infectious Diseases (ESCMID) and the International Society of Chemotherapy (ISC) for Infection and Cancer. Eur Heart J.

[12] Hill EE, Herijgers P, Claus P, Vanderschueren S, Peetermans WE, Herregods MC (2007). Abscess in infective endocarditis: the value of transesophageal echocardiography and outcome: a 5-year study. Am Heart J.

[13] Tanis W, Scholtens A, Habets J (2013). CT angiography and ^18^F-FDG-PET fusion imaging for prosthetic heart valve endocarditis. JACC Cardiovasc Imaging.

[14] Botelho-Nevers E, Thuny F, Casalta JP (2009). Dramatic reduction in infective endocarditis-related mortality with a management-based approach. Arch Intern Med.

[15] Chirillo F, Scotton P, Rocco F (2013). Impact of a multidisciplinary management strategy on the outcome of patients with native valve infective endocarditis. Am J Cardiol.

[16] Kaura A, Byrne J, Fife A (2017). Inception of the ‘endocarditis team’ is associated with improved survival in patients with infective endocarditis who are managed medically: findings from a before-and-after study. Open Heart.

[17] El-Dalati S, Khurana I, Soper N (2019). Physician perceptions of a multidisciplinary endocarditis team. Eur J Clin Microbiol Infect Dis.

[18] Davierwala PM, Marin-Cuartas M, Misfeld M, Borger MA (2019). The value of an “Endocarditis Team”. Ann Cardiothorac Surg.

[19] Nishimura RA, Otto CM, Bonow RO (2017). 2017 AHA/ACC focused update of the 2014 AHA/ACC Guideline for the Management of Patients with Valvular Heart Disease: a report of the American College of Cardiology/American Heart Association Task Force on Clinical Practice Guidelines. Circulation.

[20] Stichting werkgroep antibiotica beleid. Nieuwe SWAB richtlijnen gepubliceerd. 2019. https://swab.nl/nl/article/nieuws/490/nieuwe-swab-richtlijnen-gepubliceerd. Accessed: 3 June 2020.

[21] Nadji G, Rusinaru D, Rémadi JP, Jeu A, Sorel C, Tribouilloy C (2009). Heart failure in left-sided native valve infective endocarditis: characteristics, prognosis, and results of surgical treatment. Eur J Heart Fail.

[22] Thuny F, Beurtheret S, Mancini J (2011). The timing of surgery influences mortality and morbidity in adults with severe complicated infective endocarditis: a propensity analysis. Eur Heart J.

[23] Lalani T, Chu VH, Park LP (2013). In-hospital and 1-year mortality in patients undergoing early surgery for prosthetic valve endocarditis. JAMA Intern Med.

[24] Kang DH, Kim YJ, Kim SH (2012). Early surgery versus conventional treatment for infective endocarditis. N Engl J Med.

[25] Li JS, Sexton DJ, Mick N (2000). Proposed modifications to the Duke criteria for the diagnosis of infective endocarditis. Clin Infect Dis.

[26] Narducci ML, Pelargonio G, Russo E (2013). Usefulness of intracardiac echocardiography for the diagnosis of cardiovascular implantable electronic device-related endocarditis. J Am Coll Cardiol.

